# Pathophysiology of white matter perfusion in Alzheimer’s disease and vascular dementia

**DOI:** 10.1093/brain/awu040

**Published:** 2014-03-10

**Authors:** Rachel Barker, Emma L. Ashby, Dannielle Wellington, Vivienne M. Barrow, Jennifer C. Palmer, Patrick G. Kehoe, Margaret M. Esiri, Seth Love

**Affiliations:** 1 Dementia Research Group, Institute of Clinical Neurosciences, School of Clinical Sciences, University of Bristol, Bristol, UK; 2 Nuffield Department of Clinical Neuroscience, University of Oxford, Oxford, UK

**Keywords:** Alzheimer’s disease, brain ischaemia, cerebral blood flow, dementia, neuropathology

## Abstract

Little is known about the contributors and physiological responses to white matter hypoperfusion in the human brain. We previously showed the ratio of myelin-associated glycoprotein to proteolipid protein 1 in post-mortem human brain tissue correlates with the degree of ante-mortem ischaemia. In age-matched post-mortem cohorts of Alzheimer’s disease (*n* = 49), vascular dementia (*n* = 17) and control brains (*n* = 33) from the South West Dementia Brain Bank (Bristol), we have now examined the relationship between the ratio of myelin-associated glycoprotein to proteolipid protein 1 and several other proteins involved in regulating white matter vascularity and blood flow. Across the three cohorts, white matter perfusion, indicated by the ratio of myelin-associated glycoprotein to proteolipid protein 1, correlated positively with the concentration of the vasoconstrictor, endothelin 1 (*P* = 0.0005), and negatively with the concentration of the pro-angiogenic protein, vascular endothelial growth factor (*P* = 0.0015). The activity of angiotensin-converting enzyme, which catalyses production of the vasoconstrictor angiotensin II was not altered. In samples of frontal white matter from an independent (Oxford, UK) cohort of post-mortem brains (*n* = 74), we confirmed the significant correlations between the ratio of myelin-associated glycoprotein to proteolipid protein 1 and both endothelin 1 and vascular endothelial growth factor. We also assessed microvessel density in the Bristol (UK) samples, by measurement of factor VIII-related antigen, which we showed to correlate with immunohistochemical measurements of vessel density, and found factor VIII-related antigen levels to correlate with the level of vascular endothelial growth factor (*P* = 0.0487), suggesting that upregulation of vascular endothelial growth factor tends to increase vessel density in the white matter. We propose that downregulation of endothelin 1 and upregulation of vascular endothelial growth factor in the context of reduced ratio of myelin-associated glycoprotein to proteolipid protein 1 are likely to be protective physiological responses to reduced white matter perfusion. Further analysis of the Bristol cohort showed that endothelin 1 was reduced in the white matter in Alzheimer’s disease (*P* < 0.05) compared with control subjects, but not in vascular dementia, in which endothelin 1 tended to be elevated, perhaps reflecting abnormal regulation of white matter perfusion in vascular dementia. Our findings demonstrate the potential of post-mortem measurement of myelin proteins and mediators of vascular function, to assess physiological and pathological processes involved in the regulation of cerebral perfusion in Alzheimer’s disease and vascular dementia.

## Abstract (precis)

The pathophysiology of white matter hypoperfusion is poorly understood. Barker *et al*. quantify ante-mortem hypoperfusion by measuring myelin proteins differentially susceptible to ischaemia, and assess the extent to which vasoregulatory factors protect from or contribute to ischaemic white matter injury in Alzheimer’s disease and vascular dementia.

## Introduction

Cerebral blood flow is tightly controlled by autoregulation, in which cerebral perfusion pressure is maintained through vasomotor effectors (Paulson *et al.*, 1990), and functional hyperaemia, whereby increased energy demand in a particular region of the brain causes the release of vasoactive agents that act on local blood vessels to increase blood flow (Iadecola, 2004). Reduced cerebral blood flow as a consequence of impairment of these regulatory processes may result in cerebral ischaemia. Ischaemia is the defining pathological process in most types of vascular dementia and is also thought to contribute to the pathology and exacerbate the clinical manifestations of Alzheimer’s disease; patients with Alzheimer’s disease have reduced cerebral blood flow (Sharp *et al.*, 1986; Jagust *et al.*, 1987; Schuff *et al.*, 2009) and most show some degree of cerebrovascular pathology (Ellis *et al.*, 1996; Kalaria, 2000). Alterations in the production of factors involved in vasoregulation, such as endothelin 1 (ET1) and angiotensin converting enzyme (ACE), or those involved in angiogenesis, such as vascular enodothelial growth factor (VEGF) have the potential to contribute to chronic ischaemic brain damage and associated dementia.

ET1 is a potent and long-acting vasoconstrictor (Haynes and Webb, 1994) and thus may contribute to white matter ischaemic damage by reducing cerebral blood flow. It is cleaved from its inactive precursor big-ET1 by endothelin-converting enzymes 1 (ECE1) and 2 (ECE2) (Yanagisawa *et al.*, 2000). Both enzymes are also capable of cleaving amyloid-β (Eckman *et al.*, 2001, 2003), the deposition of which is one of the neuropathological hallmarks of Alzheimer’s disease. We previously showed the level of ET1 to be significantly raised in the temporal cortex in patients with Alzheimer’s disease compared with control subjects (Palmer *et al.*, 2012), probably in response to amyloid-β accumulation, as evidenced by *in vitro* studies (Palmer *et al.*, 2013). However, the ET1 level in the white matter has not been previously investigated; nor has the relationship between ET1 expression and ischaemia.

ACE catalyses the conversion of angiotensin I to angiotensin II, which causes vasoconstriction (Erdös and Skidgel, 1987). In the human brain, ACE protein level and activity were found to be increased in the cerebral cortex of patients with Alzheimer’s disease compared with control subjects (Miners *et al.*, 2008). ACE may also be important in Alzheimer’s disease because it is capable of cleaving amyloid-β (Hu *et al.*, 2001; Hemming and Selkoe, 2005; Oba *et al.*, 2005). ACE activity has not previously been extensively investigated in relation to white matter abnormalities or ischaemic damage. However, a recent study by Jochemsen and colleagues (personal communication) showed that increased ACE activity in the CSF was associated with increased risk of white matter hyperintensities on MRI, particularly in hypertensive patients.

VEGF has a critical role in angiogenesis, as shown both *in vitro* (Rosenstein *et al.*, 1998) and *in vivo* (Rosenstein *et al.*, 1998; Krum *et al.*, 2002). Upregulation of VEGF by ischaemia in the rat brain (Kalaria *et al.*, 1998; Sun *et al.*, 2003) is thought to be a neuroprotective response, the effect of which is to restore blood flow and prevent further tissue damage. Topical application (Hayashi *et al.*, 1998) or intracerebroventricular and intravenous infusion of VEGF after an ischaemic insult reduced infarct size and improved neurological outcome (Zhang *et al.*, 2000; Harrigan *et al.*, 2003). Post-mortem examination of human brain tissue showed expression of VEGF to be increased in the cortex in Alzheimer’s disease and vascular dementia (Kalaria *et al.*, 1998; Tarkowski *et al.*, 2002). In Alzheimer’s disease, however, VEGF was reported to bind to amyloid-β plaques, which may reduce its angiogenic activity and protective efficacy (Yang *et al.*, 2004).

We previously reported a novel way to quantify white matter ischaemic damage in post-mortem brain tissue, by comparison of the levels of two myelin proteins: myelin-associated glycoprotein (MAG), which is highly susceptible to ischaemia, and proteolipid protein 1 (PLP1), which is more resistant (Barker *et al.*, 2013). We used this method to assess the relationship between white matter injury and cerebral amyloid angiopathy (CAA) in human Alzheimer’s disease brain tissue and concluded that although CAA may lead to white matter hypoperfusion in some cases, in general it is unlikely be a major contributor. We have now investigated the relationships between ACE, ET1, VEGF and the two myelin proteins, to determine the extent to which the vasoregulatory factors protect from or contribute to white matter injury in Alzheimer’s disease, vascular dementia and control brains. We also measured factor VIII-related antigen (FVIIIRA), a protein expressed specifically by vascular endothelial cells, to assess the density of microvessels in the white matter and to relate this to the level of VEGF and severity of ischaemic damage.

## Materials and methods

### Case selection

This study had local Research Ethics Committee approval. For ACE, VEGF and FVIIIRA measurements we used the same tissue samples from the South West Dementia Brain Bank, University of Bristol (UK), on which we had previously measured MAG and PLP1 levels (Barker *et al.*, 2013) and graded CAA severity according to a five-point scale of ascending severity (zero for vessels devoid of amyloid-β up to four for vessels with severe amyloid-β deposition) as described by Olichney *et al.* (1996) (for detailed methodology see Love *et al.*, 2003). The brains had been divided midsagittally at autopsy, the left half sliced and frozen at −80°C and the right half fixed in formalin for detailed neuropathological assessment. The biochemical analyses in the present study were on samples of deep left parietal white matter from 17 cases of vascular dementia [ages 67–97 years, mean 83.4, standard deviation (SD) 7.7; post-mortem delays of 20–70 h, mean 43.7, SD 16.5], 49 cases of Alzheimer’s disease (ages 57–92 years, mean 77.5, SD 8.2; post-mortem delays of 4–72 h, mean 31.4, SD 19.3), and 33 control brains (ages 58–94 years, mean 79.6, SD 8.9; post-mortem delays of 3–67 h, mean 34.0, SD 15.9). The pathological and demographic data are summarized in Supplementary Table 1. For the ET1 measurements, a subset of these cases was used: 12 with vascular dementia (ages 67–89 years, mean 80.9, SD 6.7; post-mortem delays of 20–70 h, mean 43.1, SD 17.5), 28 with Alzheimer’s disease (ages 63–89 years, mean 76.0, SD 7.9; post-mortem delays of 4–67 h, mean 31.2, SD 20.0) of which 14 had absent to mild CAA and 14 moderate to severe CAA, and 13 control brains (ages 72–86 years, mean 76.3, SD 3.9; post-mortem delays of 12–59 h, mean 36.5, SD 13.9). The pathological and demographic data in this subset are summarized in Supplementary Table 2.

The cases with Alzheimer’s disease were selected on the basis of a diagnosis according to CERAD (Consortium to Establish a Registry for Alzheimer's disease) of ‘definite Alzheimer’s disease’ and a Braak tangle stage of V–VI (i.e. according the NIA-Reagan criteria there was a high likelihood that their dementia was caused by Alzheimer’s disease pathology). The cases with vascular dementia had a clinical history of dementia, no more than occasional neuritic plaques, a Braak stage of III or less, histopathological evidence of multiple infarcts/ischaemic lesions, moderate to severe atheroma and/or arteriosclerosis, and an absence of histopathological evidence of other disease likely to cause dementia. The normal controls had no history of dementia, few or no neuritic plaques, and no other neuropathological abnormalities.

In 48 cases, paraffin blocks were available that included the region of parietal white matter in the right cerebral hemisphere corresponding to that analysed biochemically in the left hemisphere. These were used for FVIIIRA immunohistochemistry and subsequent quantification of microvessel density, the cases comprised 12 with vascular dementia (ages 67–97 years, mean 82.8, SD 7.8; post-mortem delays of 20–70 h, mean 46.1, SD 17.6), 18 with Alzheimer’s disease (ages 63–93 years, mean 75.9, SD 9.0; post-mortem delays of 5–66 h, mean 31.4, SD 19.5) and 18 controls (ages 58–93 years, mean 77.9, SD 10.2; post-mortem delays of 12–66 h, mean 32.6, SD 15.7). The pathological and demographic data in this subset are summarized in Supplementary Table 3.

To validate our initial VEGF and FVIIIRA results we went on to analyse 74 frontal white matter samples, in which we had previously measured MAG and PLP, from the OPTIMA cohort, Thomas Willis Brain Bank, University of Oxford. Insufficient homogenate remained for measurement of ET1 level or ACE activity in these samples.

### Brain tissue

The tissue was dissected from brains that had been removed from patients within 72 h of death. The left cerebral hemisphere had been sliced and frozen at −80°C. The right cerebral hemisphere had been fixed in 10% formalin for ∼3 weeks before tissue was taken, processed and paraffin sections cut for neuropathological assessment and diagnosis. Tissue samples (200 mg) were homogenized in 1 ml 1% sodium dodecyl sulphate lysis buffer in a Precellys® homogenizer (Stretton Scientific), then aliquoted and stored at −80°C until required.

### Measurement of ET1 by sandwich ELISA

ET1 levels were measured using the QuantiGlo® Chemiluminescent ELISA kit for human ET1 (R&D Systems). Diluted brain homogenates and ET1 standard were applied to the ET1 microplate, ET1 was detected with ET1 Conjugate and Working Glo Reagent. Relative luminescence was measured using a multidetection microplate reader (see Supplementary material for details). Absolute ET1 level was measured by interpolation from the standard curve.

### Measurement of angiotensin converting enzyme activity

ACE activity levels were determined by fluorogenic activity assay using a modification of a method previously described (Miners *et al.*, 2008). An immunocapture step was incorporated to isolate ACE from brain homogenates and recombinant human ACE (R&D systems) was used to create a standard curve. ACE activity was detected with an ACE-specific substrate and fluorescence was measured at excitation/emission: 320/405 nm. The ACE-specific inhibitor captopril (Enzo Life Sciences) inhibited ACE activity by >90%. Relative ACE activity was interpolated from the standard curve (see Supplementary material for details).

### Measurement of vascular endothelial growth factor by sandwich ELISA

VEGF concentration was measured using the VEGF DuoSet ELISA kit (R&D Systems). Capture mouse anti-VEGF antibody was used to isolate VEGF from samples and VEGF standard protein was used to create a standard curve. VEGF was detected with biotinylated goat anti-VEGF antibody and peroxidase-conjugated streptavidin. Peroxidase substrate was added and the reaction stopped using Stop solution. Absorbance was measured at 450 nm and absolute protein levels were interpolated from the standard curve (see Supplementary material for details).

### Measurement of factor VIII-related antigen by dot blot

FVIIIRA level was determined by dot blot analysis, by modification of a method previously described (Ashby *et al.*, 2010). FVIIIRA was detected with a rabbit polyclonal antibody to FVIIIRA (Dako). Bound antibody was visualized with peroxidase-conjugated anti-rabbit (Vector labs) and ECL reagents (Millipore) before exposure to photographic film. ImageJ software (National Institutes of Health, Bethesda, USA) was used to measure the intensity of the dots and the relative FVIIIRA level was interpolated from the standard curve (see Supplementary material for details).

### Factor VIII-related antigen immunohistochemistry and subsequent quantification of vessel density

To determine the distribution of FVIIIRA within the white matter, paraffin sections were cut for 48 cases and immunolabelled for FVIIIRA using the general immunohistochemical method as previously described (Ashby *et al.*, 2010). Sections were incubated with the primary antibody to human FVIIIRA (rabbit polyclonal, 1:3000, Dako) overnight and bound antibody detected by a standard immunoperoxidase method (see Supplementary material for details). The areal fraction of white matter immunopositive for FVIIIRA was quantified using a Leica DM microscope and computer-assisted analysis of labelled cells in 15 randomly selected ×20 objective magnification fields per section (with the help of Image-Pro® Plus software). The mean immunopositive areal fraction was calculated and used as a measure of microvessel density.

### Immunolabelling of endothelin 1 in white matter

To determine the distribution of ET1 within the white matter, paraffin sections were cut from a small number of cases and immunolabelled for ET1 as previously described (Palmer *et al.*, 2012). The primary antibody was antibody to human ET1 (rat monoclonal 3G10 clone, 1:400, R&D Systems). In other respects, the immunohistochemical technique was the same as that for FVIIIRA.

### Statistical analysis

Relationships between the MAG:PLP1 ratio and levels of ET1, ACE activity, VEGF and FVIIIRA level across the combined Alzheimer’s disease, vascular dementia and control cohorts were assessed by Spearman’s correlation, as were the relationships between VEGF and FVIIIRA level, FVIIIRA level and FVIIIRA areal fraction, and VEGF and FVIIIRA areal fraction. Comparisons between groups were made using Kruskal-Wallis test with use of Dunn’s test for subsequent pair-wise comparisons.

## Results

### Endothelin 1

**Figure 1:**
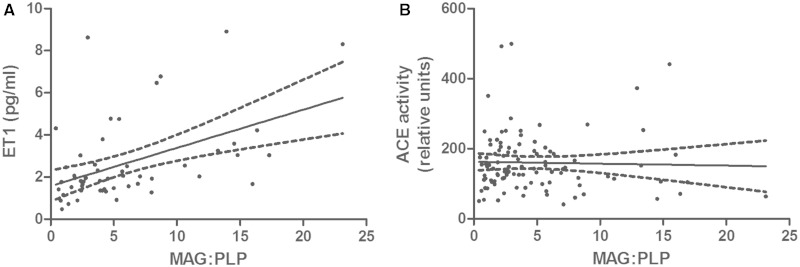
Scatterplot showing the relationship between ET1 concentration (**A**) and ACE activity (**B**) and the MAG:PLP1 ratio in the Bristol cohort. ET1 correlated positively with the MAG:PLP1 ratio (r = 0.465, *P* = 0.0005). There was no significant correlation between ACE activity and MAG:PLP.

**Figure 2:**
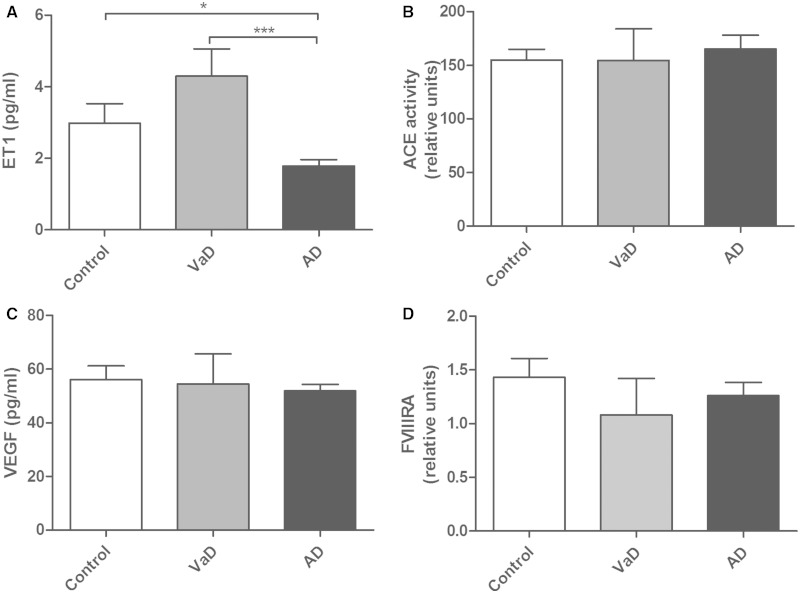
Bar charts indicating the mean levels of ET1 (**A**), ACE activity (**B**), VEGF (**C**) and FVIIIRA (**D**) in parietal deep white matter from the Bristol cohort, in control, vascular dementia (VaD) and Alzheimer’s disease (AD) brains. ET1 concentration differed significantly across the three groups (*P* = 0.0008). Dunn’s test showed significant reductions in Alzheimer’s disease compared to both controls (*P* < 0.05) and vascular dementia (*P* < 0.001). ACE activity, VEGF and FVIIIRA were not significantly altered in vascular dementia or Alzheimer’s disease compared to controls. Error bars show the SEM. *P < 0.05, ***P < 0.001.

In parietal white matter, ET1 immunolabelling was largely restricted to microvessels (Supplementary Fig. 1). White matter ET1 concentration correlated positively with the MAG:PLP1 ratio (r = 0.465, *P* = 0.0005; Fig. 1A). ET1 differed significantly between vascular dementia, Alzheimer’s disease and control brains (*P* = 0.0008; Fig. 2A); post-tests revealed that the ET1 level was significantly lower in Alzheimer’s disease than in vascular dementia (*P* < 0.001) or controls (*P* < 0.05). The mean values for ET1 concentration ± SEM were: controls 2.984 pg/ml ± 0.545, vascular dementia 4.293 pg/ml ± 0.765, Alzheimer’s disease 1.787 pg/ml ± 0.178. Mean ET1 concentration tended to decline with severity of CAA and was slightly lower in white matter from brains with moderate or severe CAA than those with absent or mild CAA but the difference was not significant.

### Angiotensin converting enzyme

White matter ACE activity did not correlate significantly with the MAG:PLP1 ratio (Fig. 1B) and was similar in Alzheimer’s disease, vascular dementia and controls (Fig. 2B). The mean values for ACE activity (relative units) ± SEM were: controls 154.9 ± 9.972, vascular dementia 154.6 ± 29.37, Alzheimer’s disease 165.3 ± 12.70. ACE activity was not affected by the severity of CAA.

### Vascular endothelial growth factor

**Figure 3:**
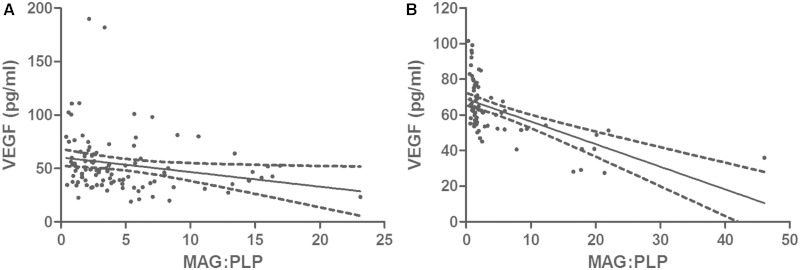
Scatterplots showing the relationship between VEGF concentration and the MAG:PLP1 ratio in the Bristol (**A**) and Oxford (**B**) cohorts. VEGF was correlated negatively with the MAG:PLP1 ratio in both cohorts (Bristol cohort: r = −0.315, *P* = 0.0015; Oxford cohort: r = −0.631, *P* < 0.0001).

VEGF concentration correlated negatively with the MAG:PLP1 ratio in both the South West Dementia Brain Bank cohort (r = −0.315, *P* = 0.0015; Fig. 3A) and the Oxford cohort (r = −0.631, *P* < 0.0001; Fig. 3B). It also correlated negatively with the ET1 level (r = −0.278, *P* = 0.0048). However, the level of VEGF was similar in Alzheimer’s disease, vascular dementia and controls (Fig. 2C). The mean values for VEGF concentration ± SEM were: controls 55.65 ± 4.724 pg/ml, vascular dementia 54.50 ± 11.21 pg/ml, and Alzheimer’s disease 51.91 ± 2.368 pg/ml. VEGF was slightly higher in white matter from brains with moderate or severe CAA than those with absent or mild CAA but the difference was not significant.

### Factor VIII-related antigen

**Figure 4:**
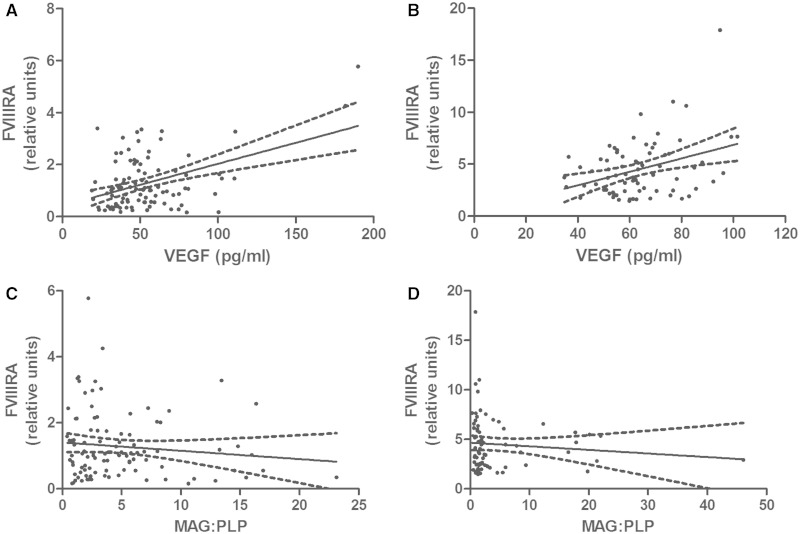
Scatterplots showing the relationships between FVIIIRA and VEGF concentration (**A** and **B**) and MAG:PLP1 ratio (**C** and **D**) in the Bristol (**A**, **C**) and Oxford (**B**, **D**) cohorts. FVIIIRA correlated positively with VEGF concentration in both the Bristol (r = 0.205, *P* = 0.042) and Oxford (r = 0.253, *P* = 0.0267) cohorts. FVIIIRA tended to decline slightly with MAG:PLP, but not significantly in either cohort.

FVIIIRA level correlated positively with VEGF concentration in both the South West Dementia Brain Bank (r = 0.195, *P* = 0.0487; Fig. 4A) and Oxford (r = 0.253, *P* = 0.0267; Fig. 4B) cohorts. FVIIIRA level tended to decline with the MAG:PLP1 ratio in both cohorts, but not significantly so (Fig. 4C and D). FVIIIRA was slightly lower in Alzheimer’s disease and vascular dementia than in controls but neither difference was significant (Fig. 2D). The mean values (in relative units) for FVIIIRA ± SEM were: controls 1.433 ± 0.176, vascular dementia 1.081 ± 0.340, and Alzheimer’s disease 1.261 ± 0.124. FVIIIRA concentration was similar across all CAA severity scores. FVIIIRA concentration was higher in controls than in cases with Alzheimer’s disease or vascular dementia but the difference was not significant (*P* = 0.0836).

### Factor VIII-related antigen level as a measure of vessel density

**Figure 5:**
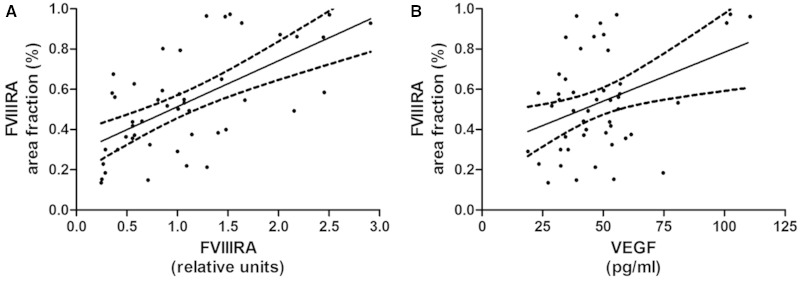
Scatterplots showing the relationships between vessel density, as measured by determining the area fraction immunopositive for FVIIIRA in histological sections of right parietal white matter, and both (**A**) FVIIIRA level and (**B**) VEGF concentration in the left parietal white matter. The vessel density correlated strongly with FVIIIRA concentration (r = 0.580, *P* < 0.0001) and tended to increase with VEGF concentration, although not significantly so. VIIIRA labelled microvessels in the white matter. Scale bar = 100 μm.

To validate the use of the FVIIIRA level in brain homogenates as a marker of vessel density, we compared FVIIIRA level with immunohistochemical measurement of vessel density in the corresponding region of white matter in the contralateral hemisphere. Immunohistochemistry with the antibody to FVIIIRA revealed the expected, strong but highly selective labelling of vascular endothelial cells in the white matter (Supplementary Fig. 1). The FVIIIRA areal fraction in the histological sections correlated strongly with FVIIIRA concentration in the contralateral parietal white matter in the 48 cases for which paired samples were available (r = 0.580, *P* < 0.0001, Fig. 5A). The FVIIIRA areal fraction in the right parietal white matter also tended to increase with VEGF concentration in the left parietal white matter, though this relationship was not statistically significant (r = 0.233, *P* = 0.112, Fig. 5B). The FVIIIRA areal fraction did not differ significantly between disease groups.

## Discussion

Although white matter ischaemic damage is thought to be an important contributor to dementia, not only in vascular dementia but also in Alzheimer’s disease, the underlying mechanisms and responses are poorly understood. Present findings suggest that the response to reduced perfusion of the white matter includes a reduction in the local production of the vasoconstrictors ET1 (but not angiotensin II), and increased angiogenesis, mediated by a rise in the level of VEGF. However, despite the severity of white matter ischaemic damage in vascular dementia, as evidenced by the decline in MAG:PLP1 (Barker *et al.*, 2013), we found rather than the expected decrease, a trend toward increased ET1, raising the possibility that ET1-mediated vasoconstriction may contribute to white matter hypoperfusion in this disease.

The positive correlation between ET1 and the MAG:PLP1 ratio suggests that as white matter perfusion falls, ET1 production also falls, presumably as a protective mechanism to reduce vasoconstriction, increase cerebral blood flow and minimize the risk of ischaemic damage. However, more severe ischaemia seems to cause a paradoxical rise in ET1. Studies in rats and rabbits showed an increase in ET1 expression immediately after ischaemia (Viossat *et al.*, 1993; Barone *et al.*, 1994; Bian *et al.*, 1994), and ET1 level rose in the plasma and CSF in the early stages of ischaemic stroke in human patients (Ziv *et al.*, 1992; Lampl *et al.*, 1997). A rise in ET1 after ischaemic stroke is not limited to the acute phase; Estrada *et al.* (1994) found that plasma ET1 level remained elevated throughout the 7-day period of their study. Present findings raise the possibility that there is an increase in the production of ET1 in the white matter in vascular dementia even in the absence an acute stroke, possibly reflecting recurrent falls in perfusion of the tissue below a critical threshold.

There is substantial literature on the upregulation of VEGF under conditions of ischaemia (Shweiki *et al.*, 1992; Liu *et al.*, 1995; Ohtaki *et al.*, 2006). This is thought to be mediated by the actions of hypoxia-inducible factor 1 alpha (HIF1A). The negative correlation between VEGF and the MAG:PLP1 ratio in cerebral white matter is likely to reflect the rise in HIF1A level and consequent increase in VEGF expression as tissue oxygenation falls. VEGF expression was shown to increase after cerebral ischaemia in rat models (Kalaria *et al.*, 1998; Sun *et al.*, 2003) and in human stroke (Issa *et al.*, 1999). This physiological response would be expected to promote angiogenesis and improve blood supply in human brain tissue, as it does in the rat (Zhang *et al.*, 2000; Sun *et al.*, 2003). That this does indeed occur is supported by our finding of a positive correlation between VEGF and FVIIIRA (a marker of vessel density) in two independent autopsy cohorts (Bristol and Oxford). We wondered whether reduced microvascular density might also contribute to white matter ischaemic damage in vascular dementia and did find that FVIIIRA level was slightly reduced in this group compared with controls but the reduction was not significant.

We have found that the changes in ET1 and VEGF levels can differ considerably between the cerebral cortex and white matter. Comparison of our current findings with those reported previously indicates that the concentration of ET1 in the cerebral cortex is approximately an order of magnitude higher than that in the white matter (Palmer *et al.*, 2012). Whereas ET1 level falls significantly in the white matter in Alzheimer’s disease, presumably as a physiological adaptation to reduced blood flow, it rises significantly in the cerebral cortex (Palmer *et al.*, 2012) and leptomeningeal blood vessels (Palmer *et al.*, 2013); as we reported previously, this is probably in response to locally elevated amyloid-β and oxidative stress. VEGF concentration is increased in the cerebral cortex and CSF in both Alzheimer’s disease and vascular dementia (Kalaria *et al.*, 1998; Tarkowski *et al.*, 2002) but it is not increased in the white matter. Our findings highlight the different mechanisms underlying ischaemic changes in the cerebral cortex and white matter and emphasize the importance of analysing these regions separately. However, it is also important to consider the interrelationships between changes in the cortex and white matter if we are to obtain an accurate picture of the pathophysiology of the cerebral circulation in the different types of dementia. Meningeal and cortical CAA contribute to white matter ischaemia, and reduced perfusion of the white matter in Alzheimer’s disease may also result from angiotensin II- and ET1-mediated constriction of perforating arterioles that traverse the cortex, but supply blood to the white matter. The lack of detectable alterations in ACE and ET1 in the white matter does not therefore mean that drugs such as angiotensin type 1 receptor antagonists and ET1-receptor antagonists might not benefit perfusion of the white matter as well as the cortex in Alzheimer’s disease (as would a reduction in meningeal and cortical CAA). Our findings also raise the possibility of benefit from ET1-receptor antagonists in vascular dementia.

Limitations of the present study include possible heterogeneity in disease severity between cerebral hemispheres, such that there may have been more or less severe vascular disease in the tissue analysed biochemically than in the contralateral, histologically assessed hemisphere. Mitigating against this are the relatively large size of the cohorts and the reproducibility of findings between the two separate cohorts, scored independently for severity of vascular disease. The correlation between the biochemically assessed level of FVIIIRA in one hemisphere and immunohistochemically assessed density of FVIIIRA-immunopositive microvessels in the other is further evidence of the approximate symmetry of vascular processes between the two hemispheres in most brains. We would also note that although the Bristol cohort was subdivided according to the presence or absence of specific clinical and pathological features, we did not make *a priori* assumptions about disease-related differences in vascular mediators, and pooled the data in the Bristol cohort, as in the Oxford cohort, when looking for interrelationships between biochemical variables and when comparing the findings in these two cohorts. One of our criteria for selection of the vascular dementia cases was that they should have minimal pathological overlap with Alzheimer’s disease; i.e. they constituted a relatively pure group of subcortical vascular dementia cases. This approach is helpful in dissecting the contribution of different pathological processes to white matter ischaemia but it should be noted that many patients have a mixed aetiology for their dementia.

In conclusion, we have shown that ET1, a vasoconstrictor, is downregulated in response to white matter hypoperfusion, whereas ACE activity is not altered. We have also shown that VEGF, a pro-angiogenic factor, is increased in response to hypoperfusion and this leads to an increase in vessel density. Presumably the downregulation of ET1 and upregulation of VEGF are protective mechanisms induced to help restore blood flow and limit further damage.

## Supplementary Material

Supplementary Data

## Abbreviations

CAA: cerebral amyloid angiopathy
FVIIIRA: factor VIII-related antigen
VEGF: vascular endothelial growth factor
