# Supplementary material for: Pathophysiology of white matter perfusion in Alzheimer’s disease and vascular dementia
Source: Brain. 2014 Mar 10;137(5):1524–32. doi: 10.1093/brain/awu040 (PMC3999715; doi:10.1093/brain/awu040)
Supplement: Supplementary Data [file supp_137_5_1524__index.html]

Pathophysiology of white matter perfusion in Alzheimer’s disease and vascular dementia — Supplementary Data 

# Pathophysiology of white matter perfusion in Alzheimer’s disease and vascular dementia

## Supplementary Data

files

**Files in this Data Supplement:**

- Supplementary Data - tif file
- Supplementary Data - docx file
