# Supplementary figures and images for: Pathophysiology of white matter perfusion in Alzheimer’s disease and vascular dementia
Source: Brain. 2014 Mar 10;137(5):1524–32. doi: 10.1093/brain/awu040 (PMC3999715; doi:10.1093/brain/awu040)

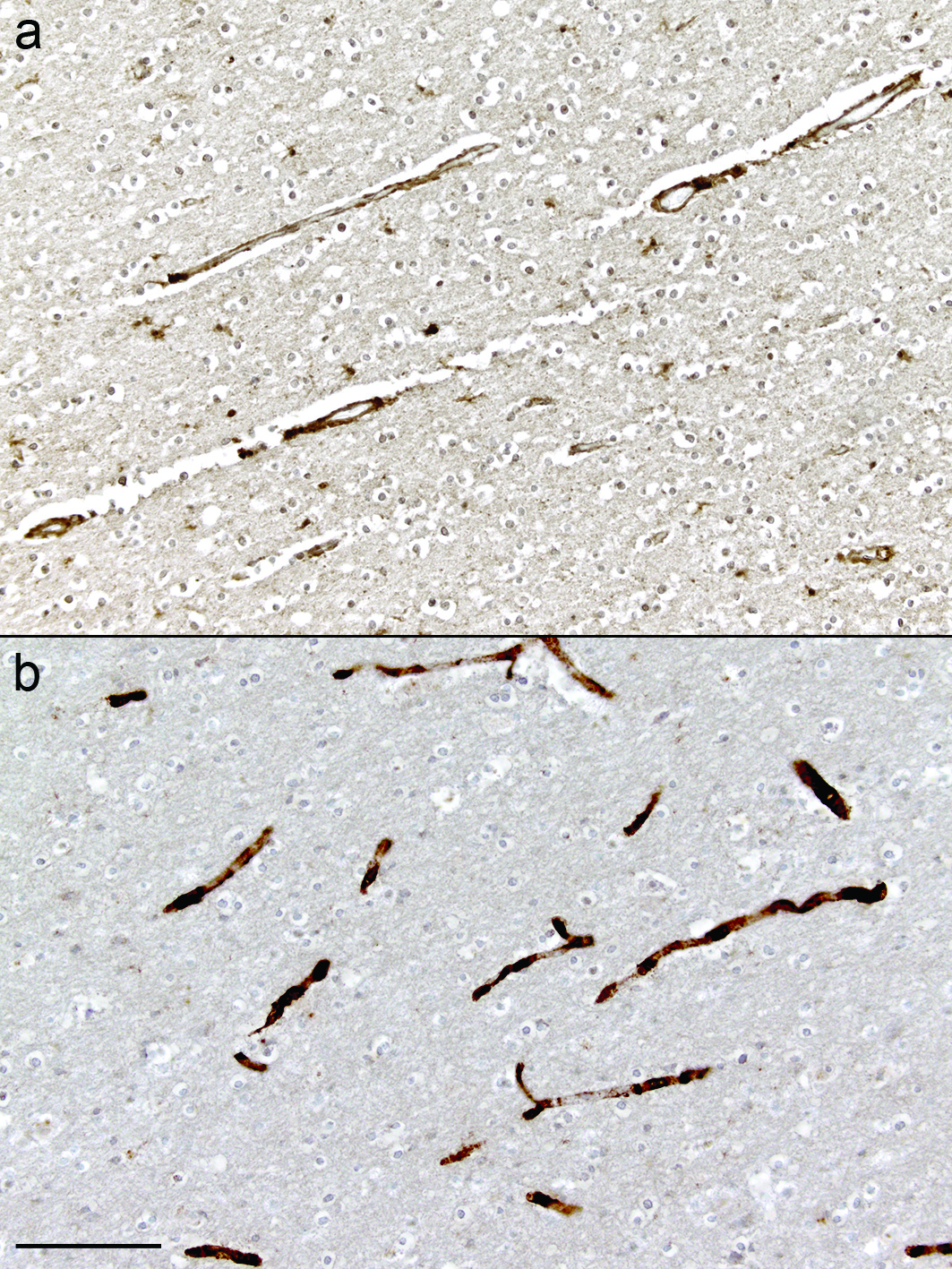

Supplement: Supplementary Data [file supp_awu040_brain-2013-01828-File010.tif]
