# Supplementary material for: Pathophysiology of white matter perfusion in Alzheimer’s disease and vascular dementia
Source: Brain. 2014 Mar 10;137(5):1524–32. doi: 10.1093/brain/awu040 (PMC3999715; doi:10.1093/brain/awu040)
Supplement: Supplementary Data [file supp_awu040_brain-2013-01828-File011.docx]

**Supplementary Methods 1**

**Measurement of ET-1**

All reagents, standards and samples were brought to room temperature before use. ET-1 standards (diluted to 0.34-83.3 pg/ml for a six-point curve), brain homogenates (diluted to 1 mg/ml total protein in Assay Diluent) and blanks were applied to the ET-1 microplate in duplicate and incubated for 1.5 h at room temperature with agitation. The plate was washed 4 times with wash buffer, tapped dry on the final wash and then ET-1 Conjugate added to each well and incubated for 3 h at room temperature on a shaker. Working Glo Reagent was prepared at this time. The plate was again washed 4 times with wash buffer, tapped dry on the final wash and Working Glo Reagent added to each well and incubated for 15 min at room temperature in the dark. Relative luminescence was measured using a multidetection microplate reader (BMG Labtech).

**Measurement of ACE activity**

Black, ninety six-well microplates (Fisher Scientific) were coated with the capture mouse monoclonal anti-ACE antibody diluted to 1µg/ml in PBS and incubated for 18 h at room temperature. After 5 washes in 0.05% Tween20 in phosphate-buffered saline (PBST), the plate was tapped dry and incubated with 1% BSA/PBS (Sigma-Aldrich) for 2 h. Meanwhile the standards and samples were prepared: seven two-fold serial dilutions of recombinant human ACE (R&D Systems) were diluted in PBS (concentration range 40 – 2500 ng/ml) and brain homogenates were diluted 1:6 in PBS. The plate was washed 5 times, tapped dry and the standards and samples loaded in triplicate along with blanks of PBS, and incubated for 2 h with agitation. The plate was again washed, and an ACE-specific inhibitor, Captopril (1mM) (Enzo Life Sciences, Exeter, UK) was added to one well in each triplicate. After 10 min, an ACE-specific substrate (Enzo) diluted 1:200 in 50 mM HEPES buffer (pH 6.5) was added to all wells and incubated at 37°C for 2 h and then at 4°C for 18 h. Fluorescence was measured in a multidetection microplate reader at Ex/Em: 320/405nm. ACE activity was inhibited by Captopril by more than 90%. Relative ACE activity was interpolated from the standard curve.

**Measurement of VEGF level**

All of the incubations were carried out at room temperature. Clear, ninety six-well microplates (Fisher Scientific) were coated with the capture mouse anti-VEGF antibody diluted to 1 µg/ml in PBS and left in the wells overnight. After 5 washes in PBST, the plate was tapped dry and incubated with 1% BSA (Sigma-Aldrich) for 1 h with agitation. Meanwhile the VEGF standard was brought to room temperature and agitated for 15 min and then the standards and samples were prepared: seven two-fold serial dilutions of the VEGF standard protein were diluted in 1% BSA/PBS (concentration range 15.6 – 1000 pg/ml) and brain homogenates were diluted 1:50 in PBS. The plate was washed 5 times, tapped dry and the standards and samples loaded in duplicate along with blanks of 1% BSA/PBS, and incubated for 2 h with agitation. After 5 washes, the plate was tapped dry and incubated with biotinylated goat anti-VEGF antibody, diluted to 100 ng/ml in PBS, for 2 h with agitation. The plate was washed 5 times then incubated with peroxidase-conjugated streptavidin diluted 1:200 in PBS for 20 min in the dark. Following 5 further washes the plate was tapped dry and 100 µl peroxidase substrate added for 20 min after which 50 µl Stop solution was added and the absorbance measured in a multidetection microplate reader (BMG Labtech) at 450 nm. Absolute protein levels were interpolated from the standard curve.

**Measurement of FVIIIRA**

Brain homogenates diluted 1:100 in 1% Tris-buffered saline (TBS) were applied, in duplicate, to a pre-wetted nitrocellulose membrane in a 96-well plate dot-blot apparatus (Bio-Rad). Seven two-fold serial dilutions of a standard reference brain tissue homogenate were also applied. The samples were allowed to drain through the membrane by gravity for 90 min, after which the membrane was washed twice in 0.05% Tris-buffered saline/Tween 20 (TBST) and incubated with 10% non-fat milk in 0.05% TBST for 1 h at room temperature to prevent non-specific binding. After 3 washes in TBST, the membrane was incubated with a rabbit polyclonal antibody to FVIIIRA (Dako, Ely, UK), diluted 1:3000 in 5% non-fat milk, for 1 h at room temperature with agitation. The membrane was washed three times in TBST then incubated with peroxidase-conjugated anti-rabbit (Vector labs), diluted 1:5000, for 1 h at room temperature with agitation. After 3 washes in TBST, ECL reagents (Millipore) were applied for 5 min, before exposure to photographic film in the dark for 1 min then development. ImageJ software (National Institutes of Health, Bethesda, USA) was used to measure the intensity of the dots and the relative FVIIIRA level was interpolated from the standard curve.

**FVIIIRA immunohistochemistry**

Sections cut at 7 μm were dewaxed and hydrated, immersed in methanol containing 3% H_2_O_2_ for 30 min, boiled in sodium citrate buffer (pH 6) and blocked in 10% normal horse serum (Vector labs, Burlingame, CA) for 20 min before overnight incubation with primary antibody diluted in PBS. Biotinylated secondary antibody (Vector Labs) was applied for 20 min, avidin-biotin horseradish peroxidase complex (VectaElite ABC, Vector Labs) for 20 min and 3,3’-diaminobenzidine containing 0.1% H_2_O_2_ (DAB, Vector Labs) for 5-10 min, before immersion of the sections in 0.16M copper sulphate and counterstaining with Harris’ hematoxylin. Finally, the sections were dehydrated, cleared and mounted. All incubations were conducted at room temperature.

**Legend for supplementary figure**

**Supplementary Fig 1.** (**a**) rat monoclonal antibody to human ET-1 and (**b**) Rabbit polyclonal antibody to F
